# Ultra-sensitive detection of mutant *KRAS* in circulating tumor DNA predicts survival in resectable pancreatic adenocarcinoma

**DOI:** 10.3389/fonc.2025.1657746

**Published:** 2026-05-04

**Authors:** Madison Cox, Amy Wells, Dominic Vitello, Larissa Masnyk, Lauren M. Janczewski, Vishvetha Rengaraju, Chengwei Peng, John Abad, Qiang Zhang, Shidong Jia, Pan Du, Akhil Chawla

**Affiliations:** 1 Department of Surgery, Division of Surgical Oncology, Northwestern University Feinberg School of Medicine, Chicago, IL, United States; 2 Northwestern Cancer Centers, Northwestern University Feinberg School of Medicine, Chicago, IL, United States; 3 Robert H. Lurie Cancer Center, Northwestern University Feinberg School of Medicine, Chicago, IL, United States; 4 Department of Medicine, Division of Hematology Oncology, Northwestern University Feinberg School of Medicine, Chicago, IL, United States; 5 Predicine, Inc., Hayward, CA, United States

**Keywords:** biomarker, circulating tumor DNA, *KRAS*, neoadjuvant chemotherapy, next generation sequencing, pancreatic cancer, ultra-deep sequencing

## Abstract

**Introduction:**

Over 90% of pancreatic adenocarcinoma (PDAC) has a *KRAS* mutation, 98% of which are single-base missense variants occurring at codon 12 (G12), codon 13 (G13), or codon 61 (Q61), with numerous studies demonstrating their clinical utility. Mutant *KRAS* has been detected in circulating tumor DNA (ctDNA) at all stages of PDAC using analysis methods such as next-generation sequencing (NGS). This study aimed to investigate the prognostic potential of *KRAS* G12, G13, and Q61 mutations present in circulating tumor DNA (ctDNA) prior to treatment initiation in localized PDAC using both standard depth (20,000x) and ultra-deep (≥100,000) sequencing.

**Methods:**

Plasma samples were collected from 45 patients with non-metastatic PDAC prior to treatment start. Baseline ctDNA was analyzed for *KRAS* G12/G13/Q61 mutations using the PredicineCARE liquid biopsy assay (20,000x sequencing depth) and the PredicineCare ULTRA assay (≥100,000x sequencing depth), providing full *KRAS* gene coverage.

**Results:**

Standard depth sequencing identified pathogenic *KRAS* mutations (G12/G13/Q61) in 11 patients. Ultra-deep sequencing demonstrated a 46% increase in detection, identifying mutant *KRAS* in these same 11 patients and an additional 7 patients deemed mutation-negative by standard assay. Presence of any *KRAS* mutation at baseline by standard sequencing was not significantly associated with overall survival (OS) (HR 2.57, 95% CI 0.94-7.04). However, detection of any *KRAS* mutation by ultra-deep sequencing in baseline ctDNA was significantly predictive of worse OS (HR 3.13, 95% CI 1.18-8.29). In both univariate and multivariate analysis, detection of mutant *KRAS* G12, Q61, or G13 in baseline ctDNA by whole-genome sequencing was superiorly predictive of worse OS over pre-therapy serum CA 19-9.

**Conclusion:**

Ultra-deep sequencing demonstrates robust detection of pertinent *KRAS* mutations that support improved prognostic stratification for patients with localized PDAC, demonstrating the potential value of advanced liquid biopsy technology to tailor treatment decisions and improve patient outcomes.

## Highlights

- Question: What is the clinical utility of employing ultra-sensitive next-generation sequencing prior to treatment initiation for analysis of mutant *KRAS* circulating tumor DNA (ctDNA) to predict survival in localized pancreatic cancer?
- Findings: In this prospective cohort study of 45 patients with localized pancreatic cancer planning to undergo curative-intent treatment with neoadjuvant chemotherapy and interval surgery, peripheral blood samples were collected prior to treatment initiation. Ultra-deep sequencing improved mutant *KRAS* ctDNA detection by 46% compared to standard-depth sequencing. Detection at diagnosis of a *KRAS* G12 mutation by standard and ultra-deep sequencing, as well as KRAS G12, Q61 or G13 mutation by ultra-deep sequencing was predictive of worse overall survival. In a multivariate analysis adjusting for age, clinical stage, and pre-therapy serum CA 19–9 level, detection of any *KRAS* mutation (G12, Q61, or G13) mutation to be a stronger predictor of worse overall survival than gold standard serum CA 19-9.
- Meaning: These findings suggest that ultra-deep next generation sequencing of circulating tumor DNA largely improves detection of mutant *KRAS* ctDNA at baseline and improves prognostic stratification and may better inform treatment decisions in patients with localized pancreatic cancer.

## Introduction

Pancreatic ductal adenocarcinoma (PDAC) has an overall five-year survival rate of just under 13%, primarily due to the majority of diagnoses being late-stage. While survival has made incremental increases yearly since 2021, it remains the third leading cause of cancer-related mortality and is projected to become the second largest cause by 2030 (1, 2). The utilization of liquid biopsy of circulating tumor DNA (ctDNA) analysis in other cancer types has provided hope for improving treatment efforts through personalization of therapy. However, there has been difficulty in applying these findings to the clinical management of PDAC, as it harbors few targetable mutations. Further efforts to identify how genomic information derived from a patient’s tumor is a necessary step to better characterize this disease and stratify patients in order to evaluate more effective treatment strategies to improve survival for the majority of patients facing a PDAC diagnosis.

Over 90% of PDAC harbors a *KRAS* mutation, making it a key biomarker of the disease. Multiple studies have identified *KRAS* mutations at all stages of PDAC, including early-stage pancreatic precursor lesions (PanIN’s), with its acquisition having been indicated as a required event for tumorigenesis (3). The majority of mutations in *KRAS* are missense variants occurring at exon 2 codon 12 (G12) with a smaller minority occurring at codon 13 (G13) or codon 61 (Q61), altogether accounting for over 95% of *KRAS* mutations in PDAC. Despite *KRAS* being mutated in over 90% of PDAC, it is considered a low-frequency mutation with studies citing a median variant allele frequency (VAF) of 0.05% (4). Detection is further challenged by low ctDNA shedding in PDAC, and dilution by non-tumor cell-free DNA (5). Therefore, it is imperative to employ incredibly sensitive sequencing technologies in order to accurately assess *KRAS* mutational status, which valuable insight on prognosis, therapy susceptibility, treatment response, and disease behavior to guide and inform management strategies aimed at cure.

Early identification of mutant *KRAS* ctDNA prior to treatment offers a noninvasive means to stratify patients based on tumor aggressiveness. Our prior work established the prognostic value of *KRAS* G12 mutations in ctDNA at baseline and after neoadjuvant therapy and surgery in resectable PDAC. We were the first to demonstrate mutant *KRAS* ctDNA as a biomarker of treatment response and poor survival, particularly in patients lacking a serum CA 19–9 response. In that study, *KRAS* G12D/V/R variants were detected in ctDNA in 13% of patients at one or more treatment time points, with baseline ctDNA present in 48% of the cohort. Baseline ctDNA presence was significantly associated with serum CA 19–9 response, and ctDNA dynamics were observed even in serum CA 19–9 non-responders, suggesting ctDNA may be a more sensitive and reliable response biomarker than the gold standard serum CA 19-9 (6). Further analyses showed that baseline presence, persistence, or acquisition of *KRAS* mutant ctDNA post-treatment predicted worse survival, with *KRAS* G12V variants associated with the poorest outcomes. Using standard-depth next-generation sequencing and digital droplet PCR confirmation, we detected mutant *KRAS* ctDNA in 86% of patients with non-metastatic PDAC in at least one timepoint from diagnosis through post-local therapy (7).

The goal of this study was to evaluate potential differences in detection capability between targeted next-generation sequencing at both a traditional sequencing depth (20,000x) and an ultra-sensitive sequencing depth (≥100,000x), as well as the prognostic potential of mutant *KRAS* G12/G13/Q61 variants detected in baseline ctDNA in non-metastatic PDAC patients undergoing curative-intent treatment.

## Materials and methods

### Study design and patient enrollment

Following institutional review board approval, 45 patients diagnosed with resectable pancreatic ductal adenocarcinoma with intention to pursue curative-intent treatment at Northwestern Medicine were prospectively enrolled. All patients had a new diagnosis of histologically confirmed PDAC and radiographic evidence of disease in the pancreatic head, body, tail or uncinate process by conventional imaging methods (e.g. CT or MRCP). Patients with active concurrent malignancy were excluded. All patients provided written informed consent. Patient plasma samples were collected between November 2022 and May 2024. All samples were de-identified at time of collection.

### Sample collection and processing

Plasma samples were drawn prior to initiation of chemotherapy for 32 patients and after either Cycle 1, 2 or 3 of neoadjuvant chemotherapy for 13 patients. All patients were treatment-naive to either regimen initiated [modified FOLFIRINOX or Gemcitabine/Nab-Paclitaxel (GnP)]. Peripheral blood (20 ml) was collected in ethylenediaminetetraacetic acid-containing tubes (cell-free DNA BCT; Streck, La Vista, NE, USA) prior to initiation of chemotherapy for 33 patients and status-post either Cycle 1, 2 or 3 for 13 patients. Two-step centrifugation of whole blood was performed. Plasma samples were processed and DNA extracted using QIAamp Circulating Nucleic Acid kits following protocol for purification of circulating nucleic acids from 2–5 mL serum or plasma (Qiagen, Redwood City, CA, USA).

### Next-generation sequencing analysis

Processed plasma was sent for next-generation sequencing with the 152-gene PredicineCARE assay or PredicineCare ULTRA assay at Predicine Inc., both CLIA-certified liquid biopsy panels (Predicine Inc., Hayward, CA, USA). The PredicineCARE Ultra assay is designed for ultra-sensitive detection of somatic mutations in circulating cell-free DNA (**Supplementary Figure S1**). The assay uses up to 60 ng of cfDNA input and follows the same library preparation workflow as the standard Predicine cfDNA assay. Enriched libraries are sequenced on an Illumina NovaSeq 6000 or NovaSeq 6000Dx/Plus platform using paired-end 2 × 150 bp reads to a target depth of 100,000x (Illumina, San Diego, CA, USA). To ensure high variant-calling specificity, a mutation is reported only when supported by two or more double-stranded mutant fragments, enabling confident detection of variants with VAF’s below 0.1%. The assay demonstrates a limit of detection (LoD_95_) of 0.075% VAF and can achieve analytical sensitivity down to 0.01% for hotspot mutations. The VAF is in the range of 0.02% to 0.1% for the Ultra Panel, while the PredicineCARE Panel only reports a VAF above 0.1%. In parallel, plasma samples were sequenced using PredicineSCORE, a low-pass whole-genome sequencing assay for detection of somatic mutations in ctDNA at 1–3x depth.

## Results

### Clinical features of study cohort

This study cohort included 45 patients with radiographically determined potentially operable PDAC who were pursuing curative-intent treatment with neoadjuvant chemotherapy followed by interval curative-intent surgery. At diagnosis, 68.8% had resectable disease (Stage IA-IIA), 15.6% had borderline resectable disease (Stage IIB), and 15.6% had locally advanced disease (Stage III). Among these, 38 patients received an mean of 5.6 cycles of neoadjuvant chemotherapy with modified FOLFIRINOX and/or Gemcitabine/Abraxane and 22 patients underwent interval surgery with Whipple procedure or distal pancreatectomy (see **Table 1** for additional clinical characteristics). Baseline ctDNA was analyzed for *KRAS* G12/G13/Q61 mutations using the PredicineCARE liquid biopsy assay (20,000x sequencing depth) and the PredicineCare ULTRA assay (≥100,000x sequencing depth with both panels providing full *KRAS* gene coverage.

**Table 1:** Clinical characteristics of patient cohort.

Characteristic	Overall (N = 45)	Resectable (Stage IA-IIA) N = 31	Borderline resectable (Stage IIB) N = 7	Locally advanced (Stage III) N = 7
Age, years				
Median	70 y.o.	71 y.o.	69 y.o.	68 y.o.
Sex, no. (%)				
Male	42%	41.9%	28.6%	57.1%
Female	58%	58.1%	71.4%	42.9%
Race, no. (%)				
Non-Hispanic White	86.7%	80.7%	100%	100%
Non-Hispanic Black	8.9%	3.2%	0%	0%
Hispanic White	2.2%	12.9%	0%	0%
American Indian/Alaskan Native	2.2%	3.2%	0%	0%
BMI (kg/m^2^)				
Mean (SD)	27.88 kg/m^2^	29.10 kg/m^2^	26.93 kg/m^2^	26.63 kg/m^2^
Smoking status, no. (%)				
Current/Former User	55.6%	64.5%	42.9%	28.6%
Never User	44.4%	35.5%	57.1%	71.4%
Type II diabetes mellitus status prior to diagnosis, no. (%)				
Yes	35.6%	38.7%	42.9%	14.3%
No	64.4%	61.3%	57.4%	85.7%
Pre-therapy serum CA 19-9 (U/mL)				
Mean (SD)	728.31	409.75	751.86	1,447
Serum CA 19–9 at/near ctDNA draw (U/mL)				
Mean (SD)	473.68	541.35	1,689.43	1,078.71
ECOG, no. (%)				
ECOG 0-1	95.6%	96.8%	85.8%	100%
ECOG 2	4.4%	3.2%	14.2%	0%
Neoadjuvant chemotherapy regimen received, no. (%)				
Modified FOLFIRINOX (mFFX)	53.3%	58.1%	42.9%	42.9%
Gemcitabine/Nab-Paclitaxel (GnP)	13.3%	16.1%	14.3%	0%
Both mFFX and GnP	6.7%	9.7%	0%	0%
5-fluorouracil + leucovorin	2.2%	3.2%	0%	0%
None	24.4%	12.9%	42.9%	57.1%
Neoadjuvant chemotherapy cycles received (mFFX or GnP)				
Mean (SD)	6.47 cycles	6.67 cycles	4.75 cycles	8 cycles
Primary tumor location				
Head	60%	71%	28.6%	42.9%
Uncinate	6.7%	3.2%	0%	28.6%
Neck	2.2%	0%	14.3%	0%
Body/Tail	6.7%	3.2%	14.3%	14.3%
Body	13.3%	9.7%	28.6%	14.3%
Tail	11.1%	12.9%	14.3%	0%
Pathological features, no. (%)				
LVSI Present	14.3%	14.3%	20%	0%
Perineural Invasion Present	67.9%	66.7%	80%	50%
Pathologic Treatment Response (CRS) ¾2	72.7% **(N = 22)**	77.8% **(N = 18)**	50% **(N = 2)**	50% **(N = 2)**
Chronic Pancreatitis	71.4%	81%	40%	50%
IPMN	28.6%	19%	40%	100%
Adjuvant chemotherapy regimen received, no. (%)				
Modified FOLFIRINOX (mFFX)	44.4%	45.2%	57.1%	28.6%
Gemcitabine/Nab-Paclitaxel (GnP)	11.1%	12.9%	14.3%	0%
Capecitabine/RT	4.4%	3.2%	0%	14.3%
Both mFFX + Capecitabine/RT	2.2%	3.2%	0%	0%
None	37.8%	35.5%	28.6%	57.1%
Adjuvant chemotherapy cycles received (mFFX or GnP)				
Mean (SD)	5.92 cycles	4.67 cycles	7.6 cycles	5.5 cycles
Detectable ctDNA *KRAS* G12/G13/Q61 mutation by PredicineCARE assay, no. (%)				
Yes	24.4%	19.4%	42.9%	28.6%
No	75.6%	80.6%	57.1%	71.4%
Detectable ctDNA *KRAS* G12/G13/Q61 mutation by PredicineCare ULTRA assay, no. (%)				
Yes	40%	35.5%	57.1%	42.9%
No	60%	64.5%	42.9%	57.1%

Significant values are bolded.

### Circulating tumor DNA mutational profile

**Figure 1:**
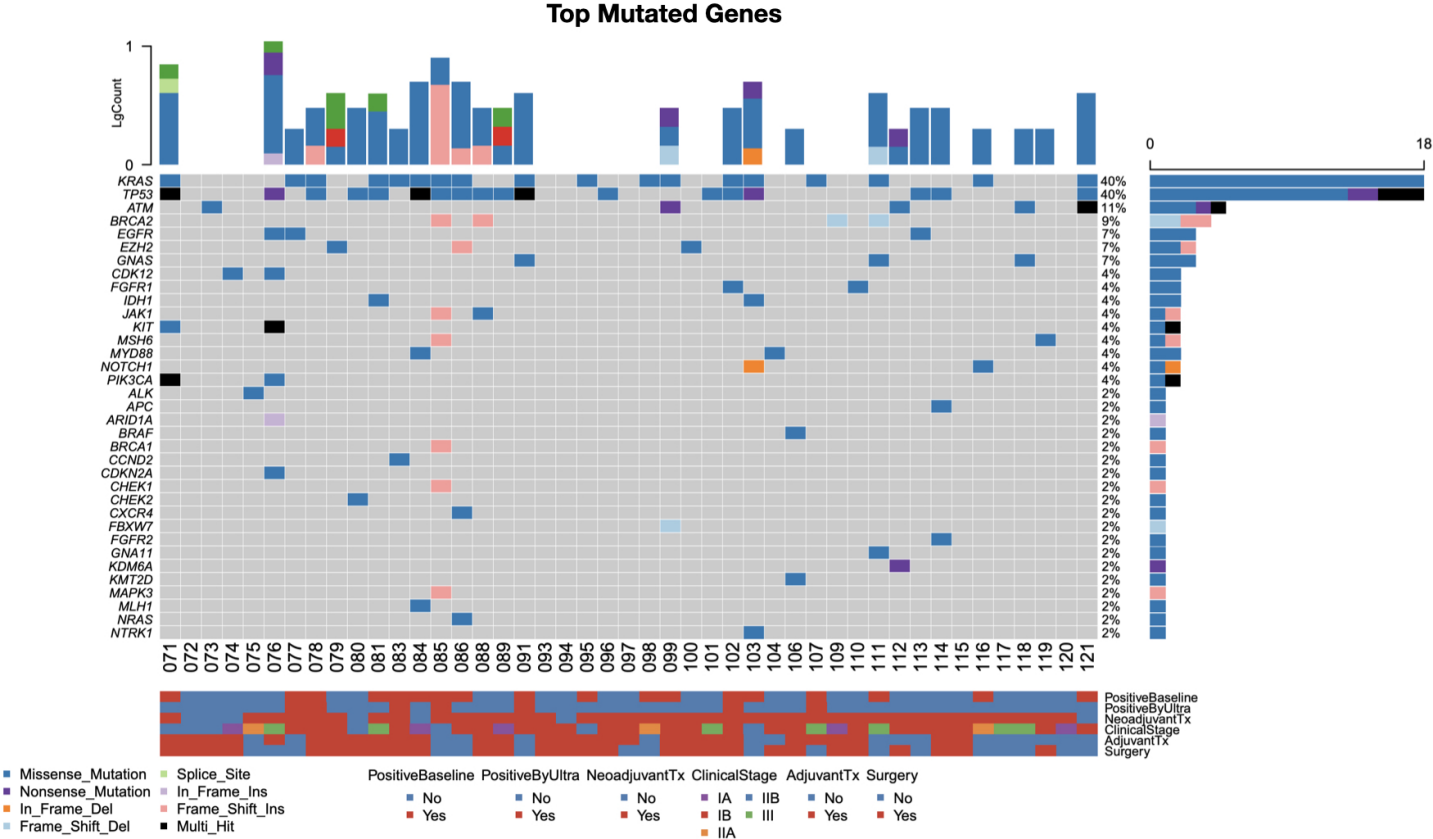
Oncoplot and gene heatmap detailing most frequently mutated genes and variation type detected by NGS and their associations with clinical characteristics. Specific genetic alterations and mutation frequency as detected by ultra-deep sequencing probes are highlighted in red.

The most common genetic mutations detected in this cohort were in *KRAS* and *TP53*, both being detected in 43% of patients, with 100% of detected *KRAS* alterations being missense variants (**Figure 1**). The standard depth assay identified pathogenic *KRAS* mutations (G12/G13/Q61) in 11 patients, of which 81.8% of mutations were glycine substitutions at codon 12 on exon 2. The ultra-sensitive assay demonstrated 100% concordance with the standard assay, detecting *KRAS* mutations in these same 11 patients as well as an additional 7 patients who were deemed mutation-negative at 20,000x depth, demonstrating a 46% increase in detection.

### Prognostic implications of KRAS ctDNA detection at baseline

**Figure 2:**
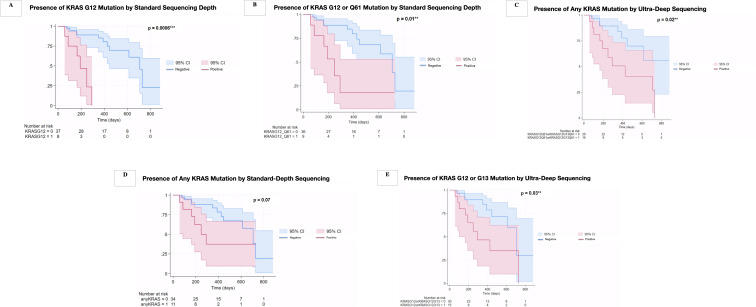
Kaplan-Meier curves demonstrating survival for each detected *KRAS* variant(s) in ctDNA by standard depth sequencing **(A–C)** and ultra-deep sequencing **(D, E)**. **(A)** Median survival for patients with a detected *KRAS* G12 in baseline ctDNA (red line) versus *KRAS* G12 negative patients (blue line) by standard depth sequencing. **(B)** Median survival for patients with detected *KRAS* G12 or Q61 mutation in baseline ctDNA (red line) versus patients negative for G12 or Q61 mutation by standard depth sequencing (blue line). **(C)** Median survival for patients with any detectable *KRAS* mutation in baseline ctDNA (red line) versus patients without any *KRAS* mutation (blue line) by ultra-deep sequencing. **(D)** Median survival for patients with any detectable *KRAS* mutation in baseline ctDNA (red line) versus patients without any *KRAS* mutation (blue line) by standard-depth sequencing. **(E)** Median survival for patients with a detectable *KRAS* G12 or G13 mutation (red line) versus patients without a *KRAS* G12 or G13 mutation by ultra-deep sequencing (blue line).

We next sought to understand if the presence of specific ctDNA mutations had prognostic significance. Source information for patient survival data, median follow-up time, and number of events was obtained from the electronic medical record. The presence of any *KRAS* mutation determined by standard assay at baseline was not significantly associated with OS, whereas detection of any *KRAS* mutation by ultra-deep sequencing was significantly associated with OS. However, detection of a *KRAS* G12 mutation in specific by standard assay in baseline ctDNA was significantly predictive of worse OS (HR 4.93, 95% CI 1.39-17.44; p = 0.006). In Notably, an additional 7 patients who had been determined to be *KRAS* negative by the standard depth assay were found to have a detectable *KRAS* G12 or G13 mutation by ultra-sensitive assay, demonstrating a 46% increase in detection by use of increased sequencing depth from 20,000x to >100,000x (11 total to 18 total patients or 24.4% to 40% of our patient cohort with mutant *KRAS* detectable in baseline ctDNA). Of the 7 patients who were *KRAS*-negative by standard assay and *KRAS*-positive by ultra-sensitive assay, five had Stage IA-IIA disease, one had Stage IIB disease, and one had Stage III disease. There was 100% inter-assay concordance demonstrated by the ultra-sensitive assay for overlapping mutations. The presence of a *KRAS* G12 mutation or Q61 mutation in baseline ctDNA by standard depth sequencing was significantly predictive of worse OS as compared to patients who were mutant *KRAS* G12 or G12/Q61 negative by standard assay, respectively (median OS 7.2 versus 22.2 months, *p* = 0.006 and median OS 14.8 versus 23.8 months, p = 0.01, respectively; **Figures 2A, B**). The presence of any *KRAS* mutation by ultra-deep sequencing was significantly predictive of worse OS as compared to patients who were *KRAS* negative (median OS 8.2 versus 23 months, *p* = 0.02; **Figure 2C**). In contrast, the presence of any *KRAS* mutation by standard-depth sequencing was not significantly predictive of survival outcome (median OS 13.8 versus 20.5 months, *p* = 0.07; **Figure 2D**). Detected *KRAS* G12 or G13 mutation in baseline ctDNA specifically by ultra-sensitive assay was also significantly predictive of worse OS as compared to those who were G12 or G13 mutation-negative (median OS 9.5 versus 22.7 months, *p* = 0.03; **Figure 2E**).

### Regressional analysis

We next sought to evaluate if the presence of specific *KRAS* ctDNA was independently associated with overall survival. In a multivariate analysis adjusting for age, sex, clinical stage and pre-therapy serum CA19–9 level, we found that the presence of a *KRAS* G12 mutation in baseline ctDNA was independently predictive of worse OS. Serum CA 19–9 level at diagnosis was also independently predictive of survival in this model, while age and clinical stage were not (**Table 2**). The presence of a *KRAS* G12 mutation in baseline ctDNA was notably more predictive compared to pre-therapy serum CA 19–9 level when adjusted for co-variates of age, sex and clinical stage (*p* = 0.005 versus *p* = 0.047, respectively; **Table 2**). The presence of *KRAS* G12 or Q61 mutation by standard sequencing was also superiorly predictive of survival than serum CA 19-9 (*p* = 0.010; **Table 2**). Most notably, the presence of any *KRAS* mutation as detected by standard-deep sequencing was found to be significantly associated with prognosis over serum CA 19–9 when adjusted for these same covariates, having not been significantly associated with worse OS in univariate analysis (*p* = 0.035; **Table 2**).

**Table 2:** Univariate analysis of demonstrating significance of age, clinical stage, pre-therapy serum CA19–9 level and *KRAS* mutational status on overall survival in resectable PDAC patients, as well as multivariate analysis demonstrating the significance of these variables when adjusted for one another.

Clinical variable	Univariate analysis		Multivariate analysis	
	HR (95% CI)	*P*-value	HR (95% CI)	*P*-value
Age*	1.04 (.97-1.12)	0.245	1.05 (0.98-1.14)	0.176
Sex				
Female	1.0 (base)	--	1.00 (base)	--
Male	.988 (.40-2.57)	0.981	0.92 (0.32-2.64)	0.882
Clinical stage				
Stage IA			1.00 (base)	**--**
Stage IB			1.9 (0.22-16.4)	0.559
Stage IIA			2.24 (0.17-28.9)	0.537
Stage IIB			0.94 (0.08-11.3)	0.962
Stage III			3.2 (0.30-34.4)	0.340
Baseline *KRAS* mutational status				
Any *KRAS* Mutation (SDS)	2.57 (.94-7.04)	0.0672	3.7 (1.1-12.5)	**0.035**
*KRAS* G12 Mutation (SDS)	7.76 (2.4-25.1)	**0.0006**		**0.005**
*KRAS* G12 or Q61 Mutation (SDS)	3.82 (1.4-10.6)	**0.010**		**0.010**
Any *KRAS* Mutation (UDS)	3.13 (1.18-8.29)	**0.022**		0.074
*KRAS* G12 or G13 Mutation (UDS)	2.92 (1.12-7.65)	**0.0291**		0.121
Pre-Therapy Serum CA 19–9 Level*	1.0 (1.0-1.0)	**0.031**	1.0 (1.0-1.0)	**0.047**

Continuous variables are notated with *.

SDS, standard depth sequencing.

UDS, ultra-deep sequencing.

Significant values are bolded.

## Discussion

We hypothesized that evaluation of ctDNA employing a highly sensitive NGS assay would be superior to a standard NGS assay for accurate *KRAS* mutational assessment in a low-shedding solid malignancy type such as PDAC, and would therefore provide improved stratification of patients with localized PDAC prior to treatment initiation. Our findings uphold well-known results that KRAS G12 mutations are highly prognostic (8–12), as we report the presence of a *KRAS* G12, Q61 or G13 mutation in circulating tumor DNA at diagnosis to be significantly predictive of worse OS in patients with non-metastatic PDAC. Furthermore, we demonstrated that *KRAS* mutational status was more predictive of overall survival than age, clinical stage, and pre-therapy serum CA 19–9 level in a multi-variate analysis (**Table 2**). Using an ultra-deep sequencing depth of >100,000x, we were able to detect mutant *KRAS* in baseline ctDNA in 32% of our patient cohort. Most notably, we found that increasing sequencing depth from 20,000x to >100,000 led to a 46% increase in mutant *KRAS* ctDNA detection. Our findings support that ultra-sensitive deep NGS provides precise results to effectively prognosticate patients with non-metastatic PDAC early in the disease course, with a large improvement in detection over standard depth NGS. In line with our results, Shut et al. in 2024 demonstrated improved detection of tumor-specific mutations in ctDNA to 88% from 67% when increasing sequencing depth to 300-500x from <300x, additionally reporting an inability to detect any tumor-specific mutations in the ctDNA of 24% of patients when using a mean coverage depth below 300x and a VAF detection cutoff of 1% (13). Takai et al. in 2015 similarly reported low detection of mutant *KRAS* cfDNA in just 8.3% patients with localized disease when using a sequencing depth of 189x, further supporting that sequencing depths below <200-300x likely fail to accurately assess *KRAS* mutational status in the majority of patients with early-stage disease (14). In a study published in 2017 as part of the Cancer Genome Atlas Research Network, a sequencing depth of ~30,000x was able to detect mutant *KRAS* in 140 of 150 patients (93%) in their cohort with a majority having localized disease, further supporting the notion that ultra-deep sequencing supports significantly improved assessment of *KRAS* mutational status in localized PDAC (15). These study findings in addition to our own support the use of ultra-deep targeted NGS liquid biopsy assay use for ctDNA analysis, as sequencing depths >30,000 demonstrate significant increases in mutation detection rate. Further improvement in NGS technologies and maturation of study findings employing ultra-sensitive liquid biopsy assays may provide further support that more patients than previously believed are ctDNA positive, potentially opening up the benefits of patient-specific care guided by individuals’ liquid biopsy results to a now significantly larger patient population. Further studies are warranted employing ultra-deep sequencing NGS assays with sequencing depths nearing 100,000x in large clinical cohorts to validate these results further and illuminate the clinical promise of ultra-sensitive liquid biopsy assays for PDAC patients at all stages of the disease.

### Prognostic significance of KRAS mutational status over serum CA 19-*9*

Notably, we demonstrated through multivariate analysis that *KRAS* mutational status by both standard and ultra-deep sequencing was superiorly predictive of overall survival over age, clinical stage, and most significantly, serum CA 19–9 level. These findings build upon our prior work where we found ctDNA levels and *KRAS* mutational status to correlate significantly with serum CA 19–9 levels and to be predictive of survival. While *KRAS* mutational status was able to be assessed by both panels, employment of ultra-deep sequencing provided the ability to include 7 additional patients in stratification, supporting its use over standard depth sequencing in order to expand the benefits of prognostication and potentially mutation-informed treatment to a larger group of patients.

### Limitations and future directions

One limitation of this study is that 29% of the patient cohort had baseline ctDNA collected after initiation of neoadjuvant chemotherapy (1–3 cycles), rather than strictly pre-treatment. Three of these patients were Stage III, one Stage IIB, and one Stage IIA, with the remainder Stage IB. The average number of neoadjuvant chemotherapy cycles received by these patients was 6.83. Because treatment itself may alter ctDNA levels and clonal dynamics, this heterogeneity could have influenced baseline mutational profiles and their prognostic associations. To assess the robustness of our findings, we performed a sensitivity analysis restricted to patients with strictly pre-treatment ctDNA samples (n = 32). In this restricted cohort, the prognostic impact of *KRAS* mutation status remained consistent, and the relative performance of standard-depth versus ultra-deep sequencing methods in risk stratification was preserved (**Supplementary Figure S1**). Future studies with larger patient cohorts should standardize baseline ctDNA collection to the pre-treatment timepoint to minimize potential confounding.

Liquid biopsy and ctDNA analysis via ultra-deep sequencing offers critical insights into tumor mutations and disease behavior, potentially enabling tailored treatment strategies and improved patient stratification in localized PDAC. Our findings support its use at diagnosis, with likely further added value from serial testing during treatment and surveillance as demonstrated in our previous works. Despite its promise, implementing ultra-sensitive NGS in the clinical management of PDAC presents notable challenges. Detecting low-frequency *KRAS* mutations, as demonstrated by this study, requires deep sequencing for high detection in localized disease, but achieving high sensitivity without compromising specificity at ultra-deep sequencing depths remains difficult. Additionally, ultra-deep sequencing carries a high cost to patients, which may unfairly limit access to such a promising methodology and personalized care to a select few. Moreover, the need for extensive datasets, complex bioinformatics, and expert interpretation increases costs and turnaround time, which are additional barriers to timely care in aggressive cancers such as PDAC. Further work to standardize ultra-sensitive NGS protocols and workflows will be essential to ensure reproducibility of results and further define liquid biopsy’s clinical utility in the management of PDAC. Creation of focused gene panels targeting key PDAC mutations such as *KRAS* may offer a practical alternative for deep sequencing that maintains high sensitivity while reducing costs, data complexity and analysis burden, with further studies needed evaluating their use. Future prospective studies should explore if treatment decisions guided by *KRAS* mutational status provide improved survival outcomes via comparative analysis to further determine the role of ctDNA as a prognostic biomarker and assess how ctDNA-guided mutation profiling may inform personalized treatment decisions by providers and improve clinical management throughout the disease course in PDAC.

## Conclusion

By enhancing baseline mutation detection and capturing tumor heterogeneity with high sensitivity, assays such as the PredicineCare ULTRA panel can improve patient stratification and inform tailored treatment strategies to potentially improve survival in localized PDAC. Emerging applications-including monitoring therapeutic response, identifying resistance mechanisms, guiding therapy selection, and enabling early detection-further highlight the potential of this technology to transform PDAC management. These findings emphasize the critical role of ultra-sensitive sequencing in refining prognostic assessment and demonstrate the value of advanced liquid biopsy approaches in delivering precise, patient-centered care.

## Data Availability

Original datasets are available in a publicly accessible repository. The original contributions presented in the study are publicly available. This data can be found here: https://www.ncbi.nlm.nih.gov/sra/PRJNA1419897.

## Supplementary material

Supplementary Figure 1 Kaplan-Meier curves demonstrating survival for each detected *KRAS* variant(s) in ctDNA in only pre-treatment samples by standard depth sequencing **(A–C)** and ultra-deep sequencing **(D, E)**. **(A)** Median survival for patients with a detected *KRAS* G12 in baseline ctDNA (red line) versus *KRAS* G12 negative patients (blue line) by standard depth sequencing. **(B)** Median survival for patients with detected *KRAS* G12 or Q61 mutation in baseline ctDNA (red line) versus patients negative for G12 or Q61 mutation (blue line) by standard depth sequencing. **(C)** Median survival for patients with any detectable *KRAS* mutation in baseline ctDNA (red line) versus patients without any *KRAS* mutation (blue line) by ultra-deep sequencing. **(D)** Median survival for patients with any detectable *KRAS* mutation in baseline ctDNA (red line) versus patients without any *KRAS* mutation (blue line) by standard-depth sequencing. **(E)** Median survival for patients with a detectable *KRAS* G12 or G13 mutation (red line) versus patients without a *KRAS* G12 or G13 mutation by ultra-deep sequencing (blue line).
