# Supplementary figures and images for: Ultra-sensitive detection of mutant KRAS in circulating tumor DNA predicts survival in resectable pancreatic adenocarcinoma
Source: Front Oncol. 2026 May 4;15:1657746. doi: 10.3389/fonc.2025.1657746 (PMC13180577; doi:10.3389/fonc.2025.1657746)

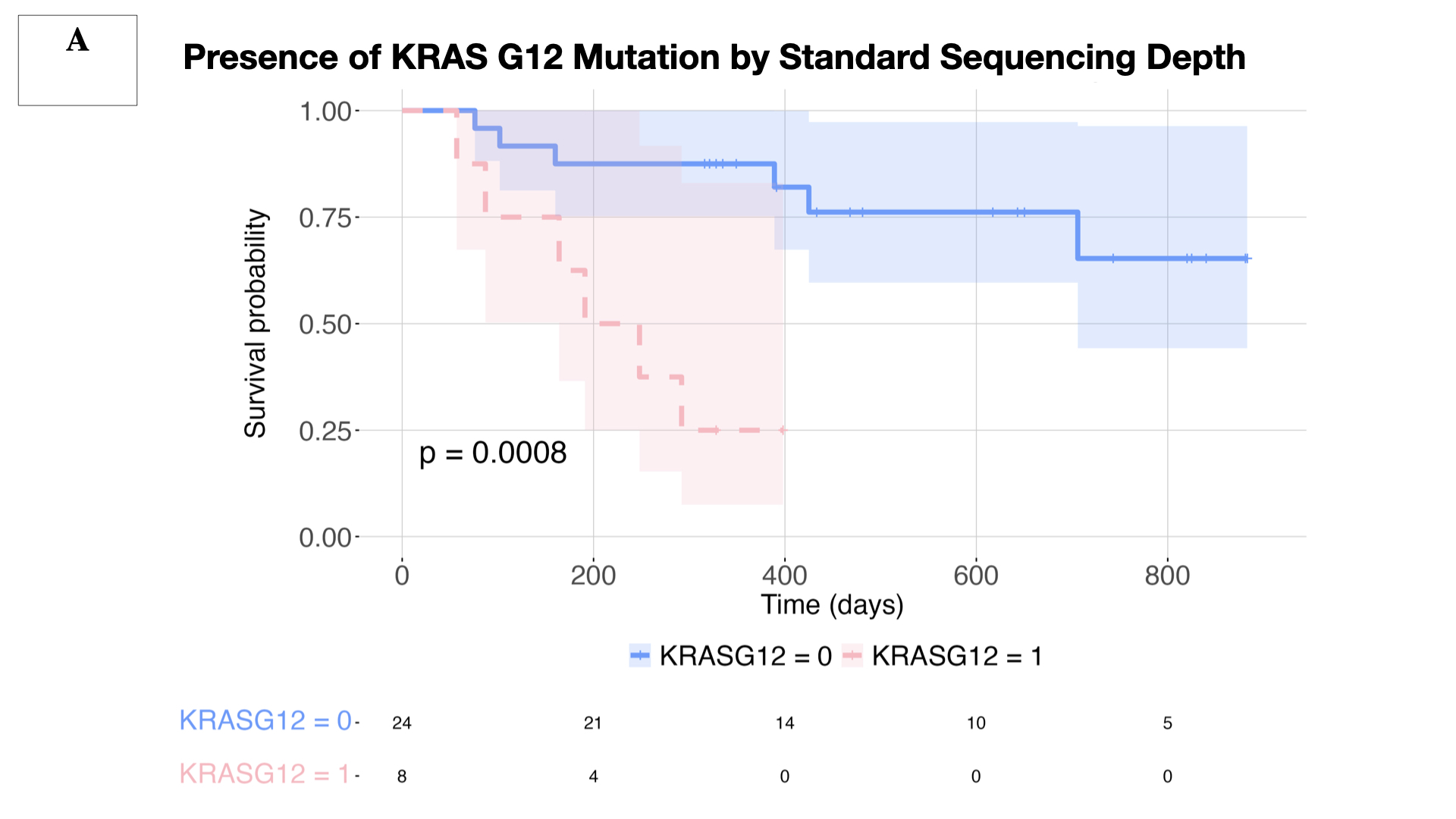

Supplement: Supplementary Figure 1 — Kaplan-Meier curves demonstrating survival for each detected KRAS variant(s) in ctDNA in only pre-treatment samples by standard depth sequencing (A–C) and ultra-deep sequencing (D, E). (A) Median survival for patients with a detected KRAS G12 in baseline ctDNA (red line) versus KRAS G12 negative patients (blue line) by standard depth sequencing. (B) Median survival for patients with detected KRAS G12 or Q61 mutation in baseline ctDNA (red line) versus patients negative for G12 or Q61 mutation (blue line) by standard depth sequencing. (C) Median survival for patients with any detectable KRAS mutation in baseline ctDNA (red line) versus patients without any KRAS mutation (blue line) by ultra-deep sequencing. (D) Median survival for patients with any detectable KRAS mutation in baseline ctDNA (red line) versus patients without any KRAS mutation (blue line) by standard-depth sequencing. (E) Median survival for patients with a detectable KRAS G12 or G13 mutation (red line) versus patients without a KRAS G12 or G13 mutation by ultra-deep sequencing (blue line). [file Image1.jpeg]

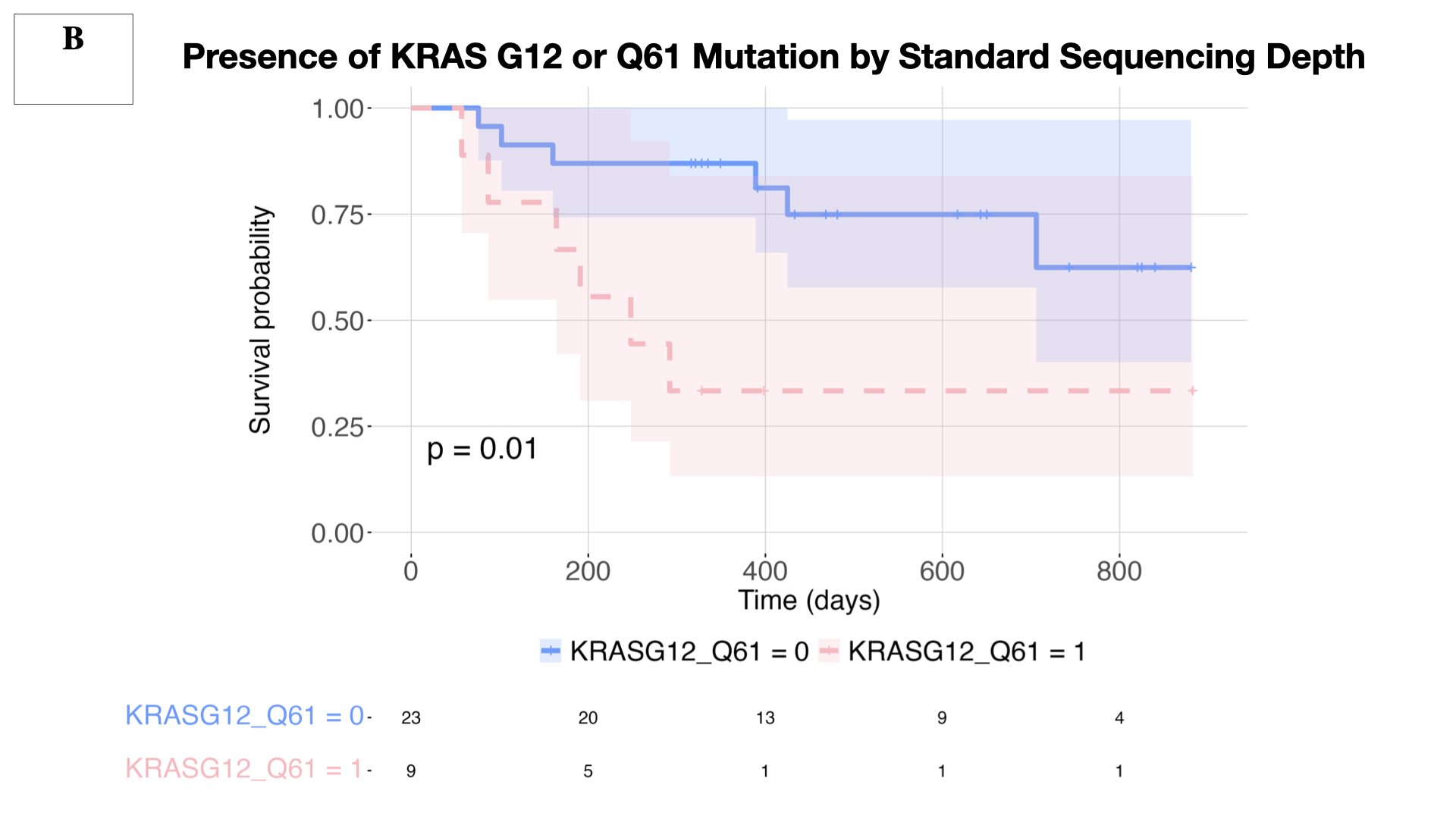

Supplement: Supplementary file 2 [file Image2.jpeg]

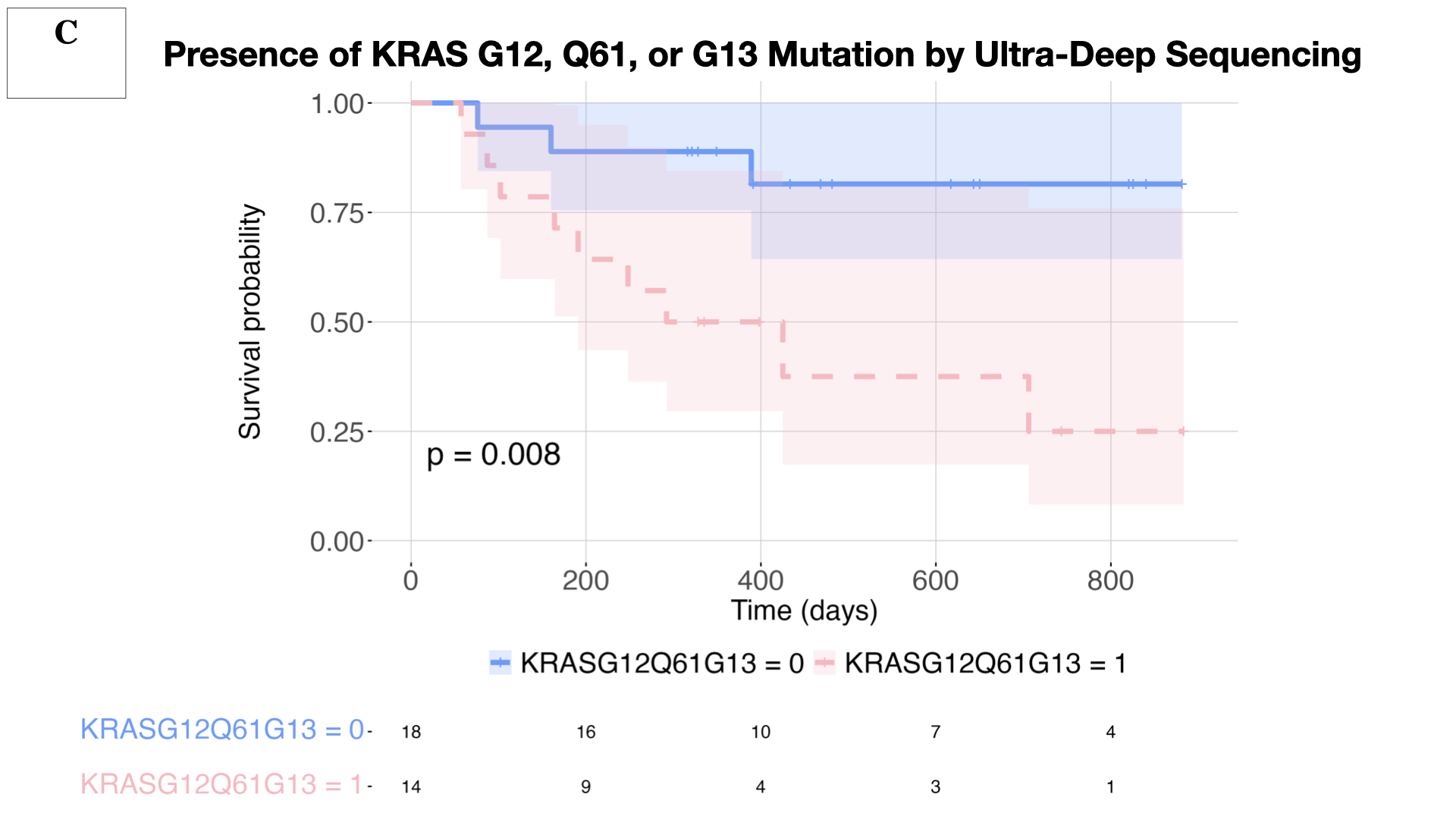

Supplement: Supplementary file 3 [file Image3.jpeg]

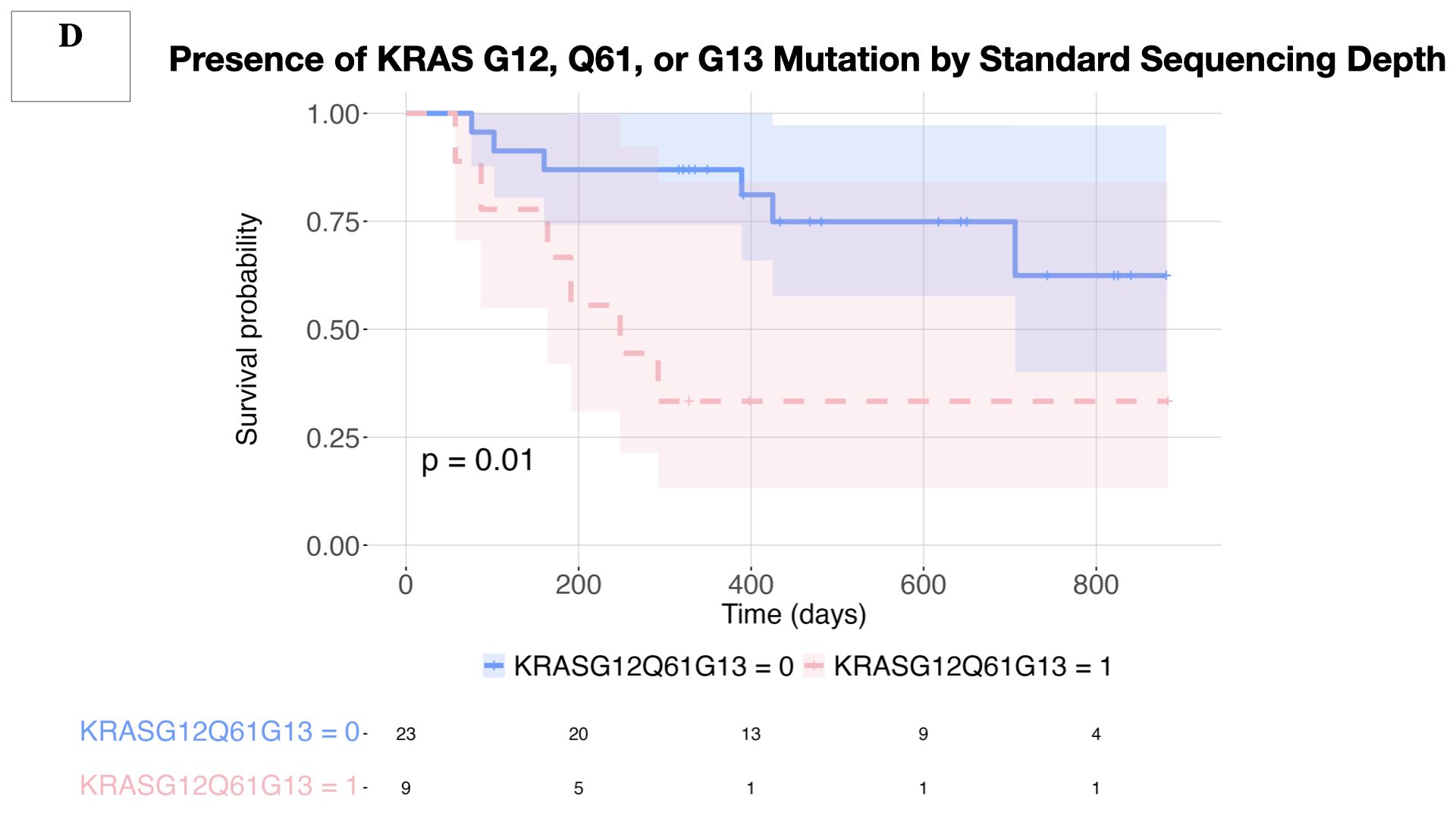

Supplement: Supplementary file 4 [file Image4.jpeg]

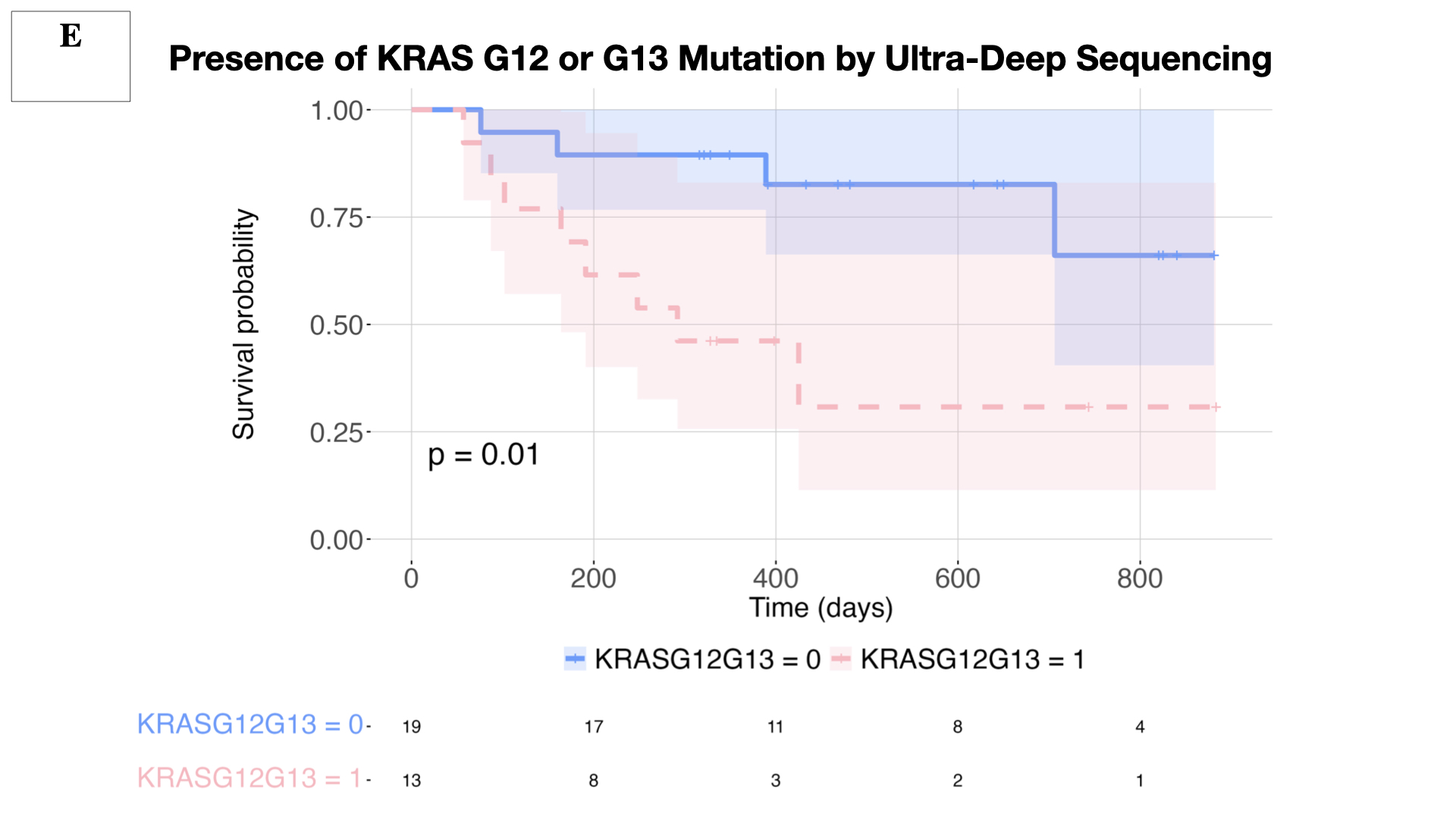

Supplement: Supplementary file 5 [file Image5.jpeg]
